# Hepatitis C RNA assay differences in results: Potential implications for shortened therapy and determination of Sustained Virologic Response

**DOI:** 10.1038/srep35410

**Published:** 2016-10-20

**Authors:** Gavin Cloherty, Stephane Chevaliez, Christoph Sarrazin, Christine Herman, Vera Holzmayer, George Dawson, Benjamin Maasoumy, Johannes Vermehren, Heiner Wedemeyer, Jordan J. Feld, Jean-Michel Pawlotsky

**Affiliations:** 1 Abbott Laboratories, Abbott Park, IL, USA; 2 National Reference Center for Viral Hepatitis B, C and delta, Hopital Henry Mondor, Université Paris-Est, Créteil, France; 3 Medizinische Klinik 1, Universitätsklinikum Frankfurt, Frankfurt am Main, Germany; 4 Klinik für Gastroenterologie, Hepatologie und Endokrinologie, Medizinische Hochschule Hannover, Hannover, Germany; 5 Toronto Centre for Liver Disease, Sandra Rotman Centre for Global Health, University of Toronto, Toronto, ON, Canada

## Abstract

Approval of Ledipasvir/Sofosbuvir for the treatment of chronic hepatitis C (HCV) includes the truncation of therapy from 12 to 8 weeks in treatment naïve, non-cirrhotic patients with baseline HCV RNA levels <6 million IU/mL (6.8 log10 IU/mL). The aim of this study was to evaluate this clinical cutoff with a different widely used commercially available HCV RNA test. Results from samples tested prospectively with Roche High Pure TaqMan HCV 2.0 test (HPS) were compared to those tested retrospectively with the Abbott RealTime HCV RNA test (ART). Using 6 million IU/mL as the cut-off, pre-treatment results were concordant in 70.4% of cases. When results with the same test measured at screening and baseline, clinical decisions could be impacted in 14.4% and 6.2% of cases for HPS and ART respectively. Using only HCV RNA cutoff of 6 million IU/mL, 29.55% of subjects would receive a different and potentially incorrect treatment duration based solely on HCV RNA test method used. A further 6–14% of subjects would have treatment decision change based on the day the sample was taken.

Worldwide it is estimated that 64–103 million people are chronically infected with the hepatitis C virus (HCV)^1^ with 3–4 million new infections per year and over 350,000 deaths due to HCV-related liver disease each year^2^. The long-term impact of chronic HCV infection is highly variable, ranging from minimal effects to chronic hepatitis, advanced fibrosis, cirrhosis, and hepatocellular carcinoma^3^.

The recent approval of all-oral direct acting antivirals (DAAs) targeting viral proteins involved in the virus life cycle deliver very high rates of sustained virologic response (SVR) with much improved side effect profiles and have revolutionized the treatment of chronic HCV^4^,^5^,^6^,^7^,^8^,^9^,^10^. These new DAAs come at a high cost which may limit the number of patients who can access them. Strategies to reduce cost are essential to increase the number of patients who can access lifesaving treatment. The recent approval of the HCV NS5A inhibitor ledipasvir (LDV) and the HCV NS5B polymerase inhibitor sofosbuvir (SOF) as a fixed-dose single-pill combination for the treatment of HCV in the US and the European Union includes the truncation of therapy from 12 to 8 weeks in treatment naïve, non-cirrhotic patients with HCV RNA levels <6 million IU/mL (6.8 log_10_ IU/mL) based on the findings of the ION-3 trial^11^. This approach has been adopted by the US FDA and, with reservations, the European guidelines^12^,^13^. The HCV viral load data from the trials used to define this clinical cutoff were generated using a single test, the manual Roche High-Pure-System/COBAS® TaqMan assay (HPS, Roche Molecular, Pleasanton, CA, USA). This manual assay is not widely used in most countries where anti-HCV therapies are prescribed. Previous studies have shown differences in performance between this manual method and other automated methods more widely used in clinical practice^14^,^15^,^16^. Investigators have reported differences in detection rates between HPS and Abbott RealTime HCV RNA test (Abbott Molecular, Des Plaines IL., USA) at the end of treatment (EOT) with DAA regimens and contrary to past experience with interferon-containing treatments, low levels of quantifiable HCV RNA at EOT do not preclude treatment success^17^. In a recent analysis presented at the International Liver Congress 2016 the authors show that 8 weeks of LDV/SOF resulted in SVR rates of 97% in multiple, large, real-world cohorts, comparable to the SVR rates seen in ION-3 post hoc analysis^18^. More recently investigators have evaluated the applicability of this baseline viral load threshold in clinical practice with two commercially available automated tests^19^.

In this study we evaluate this new clinical cutoff (6 million IU/mL) used to shorten LDV/SOF therapy. A secondary aim of this study is to investigate the performance of highly sensitive, automated, commercially available HCV RNA viral load assays and the impact of detecting low levels of HCV RNA at EOT and post treatment week 12.

## Methods

**Figure 1:**
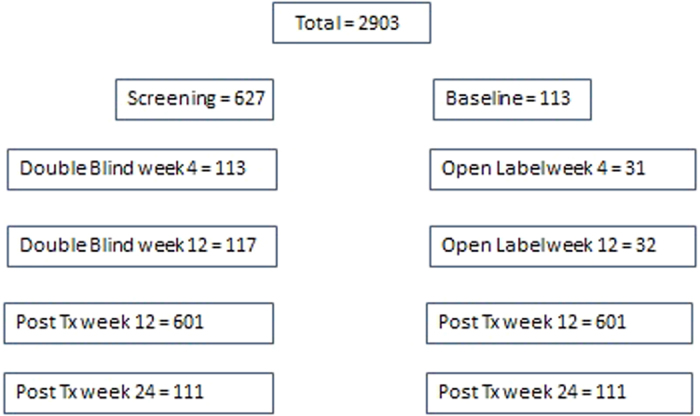
Breakdown of samples tested with both Roche High Pure HCV RNA 2.0 and Abbott RealTime HCV RNA tests by timepoint.

A total of 2903 samples from 631 subjects who enrolled in AbbVie’s SAPPHIRE I Phase III trial were included in this study (Fig. 1). SAPPHIRE I is a randomized, double-blind, placebo-controlled trial (NTC 01716585) which evaluated the all oral combination of 3 direct acting antivirals (3D) protease inhibitor paritaprevir with ritonavir and the NS5A inhibitor ombitasvir plus the nonnucleoside polymerase inhibitor dasabuvir and ribavirin in previously untreated, non-cirrhotic, patients with HCV genotype 1 infection. In this trial 473 subjects were enrolled in the “Double Blind” arm and received 12 weeks of the study drug combination. A further 158 subjects enrolled in the “Open Label” arm and received 12 weeks of placebo followed by 12 weeks of study drug combination. The results reported in this study reflect the treatment arm and the time of sampling e.g. Open Label week 4. All the patients provided written informed consent. The study was conducted in accordance with the International Conference on Harmonisation guidelines, applicable regulations, and the principles of the Declaration of Helsinki. The study protocol was approved by the independent ethics committee or institutional review board at each study site. All samples were tested prospectively with HPS, samples were stored at −80 °C and results were compared to those tested retrospectively with ART according to the manufacturer’s instructions. Details of the assay characteristics have been described previously^19^,^20^.

To evaluate the potential impact of quantitative viral load bias reported by HPS and ART around the 6 million IU/mL clinical cutoff, the results from pre-treatment samples with values for both methods (n = 741) were compared for concordance. These samples represented 627/631 from screening and 113/631 from baseline. The interval from Screening to Baseline in this study averaged 23 days with a minimum of 7 days, a maximum of 92 days and an overall standard deviation of 12.88 days. The potential impact of natural viral fluctuations prior to the initiation of treatment was explored where possible, by comparing results from subjects sampled at screening and baseline with the same viral load method for concordance at the 6 million IU/mL cutoff. Screening and baseline viral load results tested with the same method were available for 631 subjects for HPS and 113 for ART. The number of data points available for analysis with ART was lower due to insufficient residual sample volume. To understand how viral loads measured by these two methods and any bias between them vary over the dynamic range of both assays, correlation was measured at key intervals and the data adjusted for the bias was plotted and a linear regression performed. To investigate differences in detection at the low end of the assay dynamic range, samples from all subjects and timepoints with sufficient volume were tested with ART (n = 2903) and concordance measured against the test of record, the HPS method, with 25 IU/mL and 100 IU/mL as the cutoff for detection, respectively.

### Statistical analyses

All data were analyzed with PC SAS 9.3 (SAS Institute Cary, North Carolina, USA). A least squares linear regression was performed to examine the association between the HPS assay result (log IU/mL) and the Abbott RealTime HCV result (log IU/mL). The data were plotted and the Pearson correlation coefficient, the coefficient of determination, the point estimates for the slope and intercept of the regression line were calculated. Additionally an analysis of the bias between the two assays was performed. For each specimen, the bias was defined as the difference between the ART HCV and HPS results. The summary statistics of the difference were calculated (mean, and standard deviation) along with their confidence limits. A *t* test was performed to test whether the mean bias was statistically different from zero. P values <0.05 were considered to be statistical significant. In order to visualize the differences between the two assays a Bland-Altman bias plot of the bias versus the average value of the two assays was generated.

### Study Oversight

All patients provided written informed consent relevant to the use of the current study.

## Results

**Figure 2:**
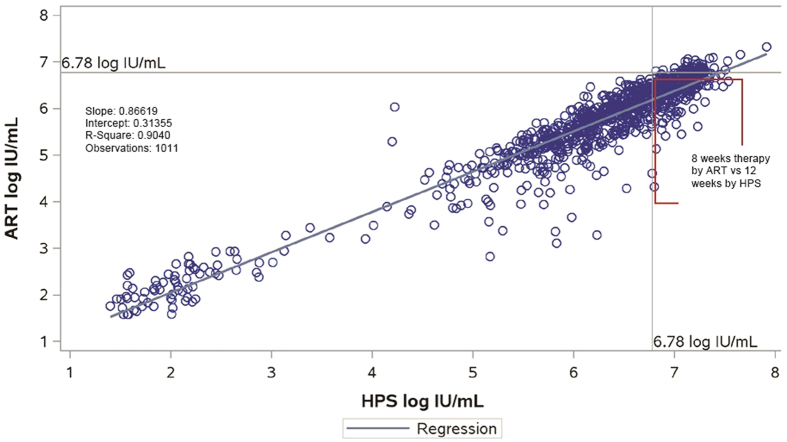
Least-Squares regression between Abbott RealTime HCV vs Roche HPS/TaqMan 2.0 for all data points within the dynamic range of both assays. Clinically significant discordant results are found in red box.

**Figure 3:**
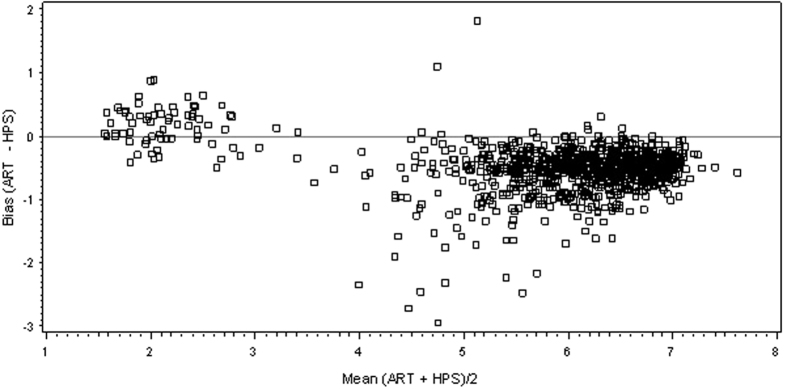
Bland Altman Bias Plot Abbott RealTime HCV vs Roche HPS/TaqMan 2.0 for samples with viral load 25 IU/mL or Greater (n = 1011). The mean difference between the two assays was −0.50 (95% Confidence Interval: −0.53 and −0.48; p < 0.0001) log_10_ IU/mL HCV RNA.

**Figure 4:**
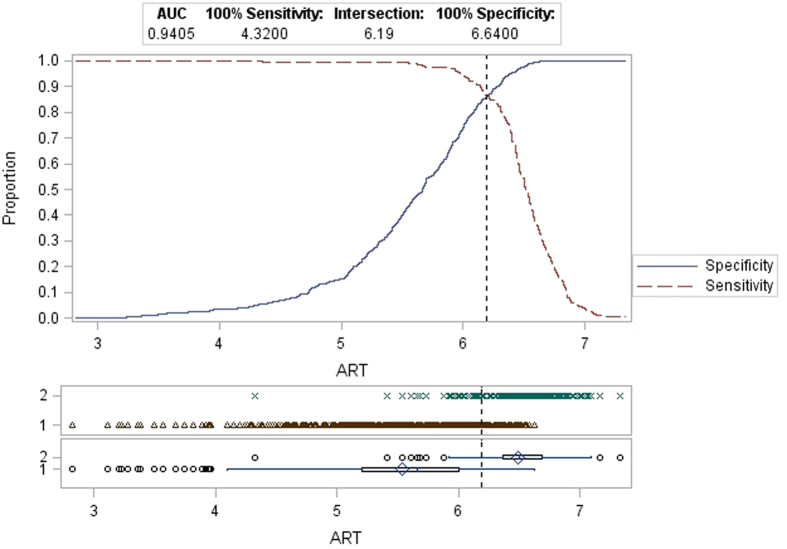
ROC analysys of all available pre-treatment viral load measurements with both Roche HPS/TaqMan 2.0 HCV and Abbott RealTime HCV assays. For this analysis Roche HPS/TaqMan 2.0 HCV was considered gold standard.

A total of 2903 paired HCV RNA level results from the 631 patients enrolled in the study were obtained. There were 1011 specimens with detectable HCV RNA results that were within the dynamic range for both tests; significant correlation between the 2 measurements was observed (r = 0.9507, p < 0.0001 Fig. 2). The mean difference between the two assays was −0.51 (95% Confidence Interval: −0.53 and −0.48; p < 0.0001) log_10_ HCV RNA IU/mL. A Bland Altman plot analysis is shown in Fig. 3. Of the 741 pre-treatment samples tested with both HPS and ART, using 6 million IU/mL as the cut-off, results were concordant in 522 (70.4%) and discordant in 219 (29.6%) of cases. Discordance by time point testedwas 28.9% vs 29.7% respectively for baseline and screening. Of note all discordant results were HPS >6 million and ART <6 million with a mean positive bias at this cutoff of 0.61 log IU/mL (95% Ocnfidence Interval −0.64 to −0.57 ; p < 0.0001). When ROC analysis was performed on the same dataset with HPS as the gold standard the intersection of the sensitivity (>6 M) and specificity (≤6 M) is 6.19 log IU/ mL (Fig. 4).

When viral load results generated with the same test measured at screening and baseline (n = 631 for HPS and 113 for ART) were compared, clinical decisions (treatment duration) could be impacted in 14.4% cases for HPS (8.1% extended and 6.3% truncated) and 6.2% for ART (3.5% extended and 2.6% truncated). The mean difference in viral load between these two time points with the same test was between 0.07 log IU/mL and −0.02 log IU/mL for HPS and ART respectively.

**Figure 5:**
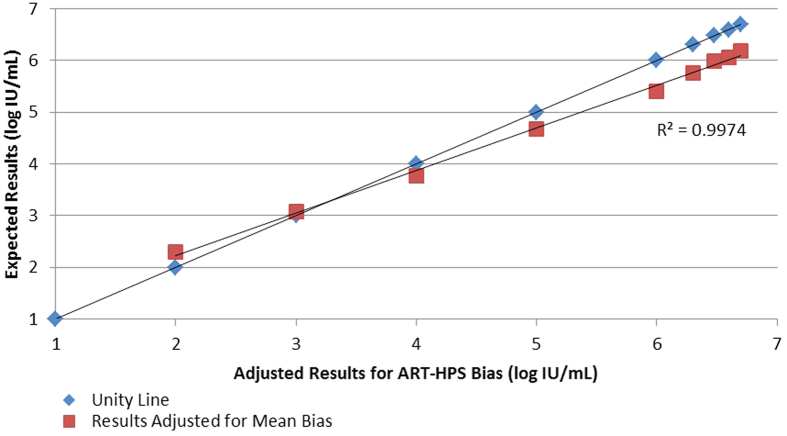
Samples with Roche HPS/TaqMan 2.0 HCV results falling within log intervals of the assays dynamic range were adjusted based on the calculated negative bias observed with the Abbott RealTime HCV test. The linear regression of mean expected results compared to the mean expected results adjusted for bias.

**Figure 6:**
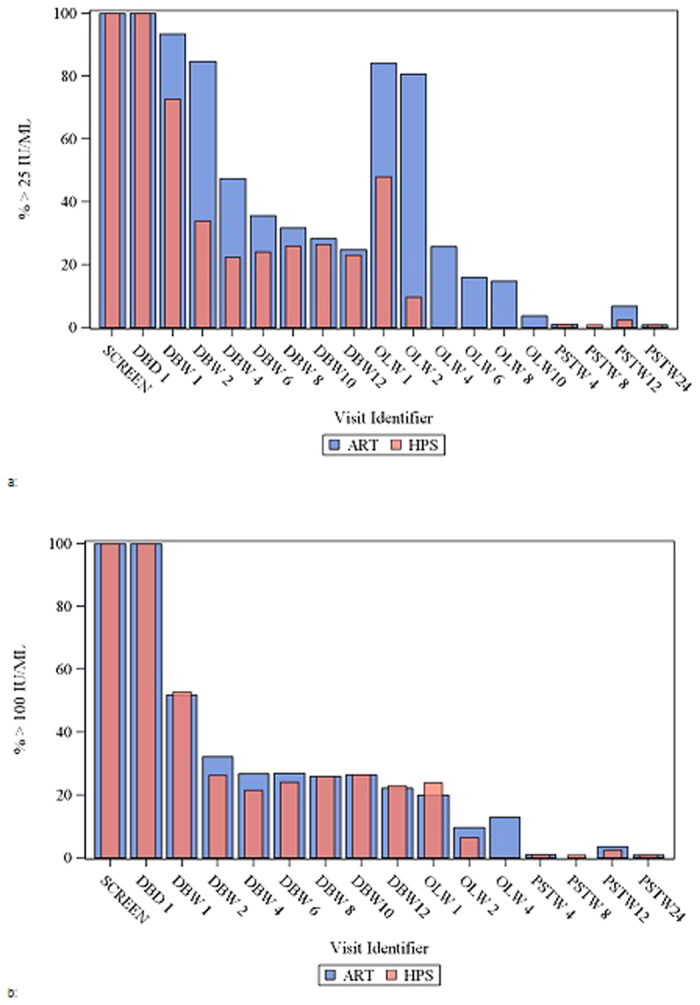
(**a**) Percent detected by Abbott RealTime HCV (ART) and Roche HPS/TaqMan 2.0 HCV using 25 IU/mL as cut-off. DBD = Double Blind Day; OLW = Open Label Week; PSTW = Post Treatment Week. (**b**) Percent detected by Abbott RealTime HCV (ART) and Roche HPS/TaqMan 2.0 HCV using 100 IU/mL as cut-off. DBD = Double Blind Day; OLW = Open Label Week; PSTW = Post Treatment Week.

The correlation between ART and HPS measured at various intervals accross the dynamic range of HCV viral loads seen in this study revealed that the ART-HPS bias went from +0.30 log IU/mL for viral loads <2 log IU/mL (n = 28) to −0.56 log IU/mL at viral loads greater than 6.6 log IU/mL (n = 428). The linear regression of the data adjusted for mean bias at key viral load intervals can be seen in Fig. 5. Using 25 IU/mL as the cutoff for detection, the percent concordance at Double Blind Week 4 was 73.3% (n = 116) with 30 of 31 discordant results detected by ART but not by HPS. The level of agreement increased dramatically to 98.3% (n = 117) at Double Blind Week 12 time point with only two discordant results detected by ART but not HPS. Analysis of the Open Label results demonstrate similar results with 74.2% agreement (n = 31) with all 8 discordant results detected by ART and not HPS. Agreement at Open Label Week 12 (EOT) was 100% (n = 32). Interestingly, at Post Treatment Week 12 (n = 601) time point using 25 IU/mL as the cutoff, 95.7% of samples were undetectable by both methods and 4.3% (n = 26) samples were detectable with ART and not detected by HPS (Fig. 6a). In a similar analysis but using 100 IU/mL as the cutoff for detection the percent concordance at Double Blind Week 4 rose to 94.8% (n = 116) with all 6 discordant results detected by ART but not by HPS. Only one sample gave a discordant result using this cut off at Double Blind Week 12 and this sample was detected by HPS but not by ART. The level of concordance also increased in the Open Label results using a higher cut-off with only 4 discordant results (12.9%) at week 4 and 100% concordance at Week 12. Post Treatment Week 12 had 1.3% (n = 8) samples detected with ART and not detected by HPS (Fig. 6b). Regardless of the cut-off used, concordance between the two methods is 100% at Post Treatment Week 24 (n = 111).

## Discussion

The approved label for sofosbuvir/ledipasvir indicates that treatment naive non-cirrhotic patients with a baseline viral load <6 million IU/mL are eligible to short therapy of 8 weeks while all others should receive a full 12 weeks of therapy^12^,^13^. As seen previously with the establishment of on-treatment response-guided therapy rules, no attention was paid during the establishment of this treatment truncation rule to the different performance characteristics of commercially available viral load tests and how they compare to the single, manual method used in clinical trials but not widely used in clinical practice. The data from this study demonstrated good agreement between the manual HPS and automated ART methods for the quantification of HCV RNA in genotype 1 patients. As seen in previous studies the bias seen between HPS and ART appears to flip with HPS higher than ART in high viral load samples whereas ART was shown to be the more sensitive assay with consistently higher quantitative HCV RNA results in samples with very low titres around the lower limit of quantification^20^,^21^,^22^,^23^,^24^. This may be due in part to differences in assay calibration strategies employed by the two methods. HPS uses an internal quantitation standard (IQS) to calibrate viral load results. The IQS cannot adjust for variances at both the upper and lower end of the dynamic range. Other studies have described that CTM results are not linear across the entire dynamic range and require the use of a third order polynomial linear regression (y = ax3 + bx2 + cx) with ±0.2 log IU/mL allowable maximum difference from linearity^25^,^26^,^27^. In contrast, the ART assay employs a simple linear function (y = ax + b) using a two-point external calibration strategy to quantitate viral load. The ART assay has been shown in studies to be linear across the entire dynamic range of the test^28^,^29^. Therefore, it is possible that a contributing factor to the observed discordance is the calibration strategy employed by each assay.

In this study, using only HCV RNA cutoff of 6 million IU/mL, 29.55% of subjects who would be eligible for treatment truncation based on the HPS results would not have been eligible based on the ARTresults. A further 6–14% of subjects would have treatment decision change based on the day the sample was taken due to natural fluctuations in pretreatment viral loads. When analyzing these natural fluctuations it should be noted that there were greater number of cases tested at both time points by HPS than by ART which may have some impact on the differences seen between methods.

The results of this study also support published findings of other investigators that the ART HCV RNA assay is a more sensitive test and detects virus in a higher number of patients on treatment, that the positive bias in quantitation between ART and HPS will more likely result in a quantifiable result at the low end of the dynamic range with ART and that the number of concordant undetectable results significantly increases with time on treatment^15^,^16^,^17^,^18^,^19^,^20^,^21^,^22^,^23^,^24^,^25^,^30^. With previous less potent regimens a detectable on-treatment viral load particularly at the end of therapy might imply a patient was failing therapy and/or treatment extension might be considered. Our findings also support that a positive, even quantifiable HCV RNA result, at end of treatment does not preclude achievement of a sustained virological response with these new 3-D regimens and do not warrant modification or extension of therapy^17^. Given that these inherent differences between assays used in clinical trials from those used in routine practice may not be appreciated by clinicians, the finding of more positive tests using ART at early time points following the end of therapy is noteworthy. Concordance was 100% at week 24 post-treatment, which may argue for re-testing all samples with detectable low level viremia detected at 12 weeks post-treatment at this later time point, particularly if using the ART assay.

In this study, using only HCV RNA cutoff of 6 million IU/mL, 29.55% of subjects who would be eligible for treatment truncation based on the HPS results would not have been eligible based on the ART results. A further 6–14% of subjects would have the treatment decision changed based on the day the sample was taken due to natural fluctuations in pretreatment viral loads. The patient profile of subjects enrolled in SAPPHIRE I and the number of subjects with baseline viral loads <6 million IU/mL using HPS (64.5%) compares very closely with the ION-3 registration trial (59%) used to establish this treatment truncation rule. It will be important to follow-up on real-life data that are based on ART and other automated platforms such as the COBAS® AmpliPrep version COBAS® TaqMan assay (CAP/CTM, Roche Molecular, Pleasanton, CA, USA) measurements to determine whether the potentially higher number of patients eligible for shortened treatment durations will result in a higher risk for relapse. The mean bias around the 6 million IU/mL clinical cutoff of 0.61 log IU/mL observed in this study would imply that a viral load of between 1.5 and 2.0 million IU/mL with ART (6.18–6.30 log IU/mL) would be equivalent to 6 million with HPS (6.78 log IU/mL). This threshold is supported by ROC analysis using all 741 available pre-treatment data points and HPS as truth/gold standard where the intersection of the sensitivity (>6 M) and specificity (≤6 M) is 6.19 log IU/ mL. In another study comparing CAP/CTM with ART the bias at 6.78 log IU/mL was smaller (0.41 log IU/mL vs 0.61 log IU/mL) which clinically significant diferences also exist between the manual version of the test used in the clinical trials (HPS) and the automated versions widely used in the field (CAP/CTM)^30^. Although insufficient sample volume as available for additional testing in this study investigations should be conducted to translate the threshold from the clinical trials (HPS 6.78 log IU/mL) to those reported by the automated CAP/CTM for patient management.

Current guideline recommendations based on an absolute quantitative baseline HCV RNA value derived from a single test not widely used in clinical practice may be inappropriate and may result in misclassification with regard to this clinical cutoff. At the minimum, ranges of HCV RNA levels should be used and ideally stated for all commercially available assays.

The findings of this study would supports those of other investigators which demonstrated limited or no utility for very sensitive, quantitative viral load testing at baseline to inform treatment duration and at end of treatment as an indicator of subsequent SVR^17^,^29^. However given the widespread use of HCV RNA testing, it serves as a useful tool for the confirmation of active infection treatment adherence and determination of SVR.

Limitations of this study include the fact that ART results were generated retrospectively and that there were greater number of cases tested at both time points by HPS than by ART.

## Additional Information

**How to cite this article**: Cloherty, G. *et al*. Hepatitis C RNA assay differences in results: Potential implications for shortened therapy and determination of Sustained Virologic Response. *Sci. Rep.* **6**, 35410; doi: 10.1038/srep35410 (2016).
